# Synthetic Biomimetic Membranes and Their Sensor Applications

**DOI:** 10.3390/s120709530

**Published:** 2012-07-11

**Authors:** Young-Rok Kim, Sungho Jung, Hyunil Ryu, Yeong-Eun Yoo, Sun Min Kim, Tae-Joon Jeon

**Affiliations:** 1 Institute of Life Sciences and Resources & Department of Food Science and Biotechnology, Kyung Hee University, Yongin 446-701, Korea; E-Mail: youngkim@khu.ac.kr; 2 Department of Biological Engineering, Inha University, Incheon 402-751, Korea; E-Mails: sunghomail@gmail.com (S.J.); hyunil.ryu@gmail.com (H.R.); 3 Nano-Mechanical Systems Research Division, Korea Institute of Machinery and Materials, Daejeon 305-343, Korea; E-Mail: yeyoo@kimm.re.kr; 4 Department of Mechanical Engineering, Inha University, Incheon 402-751, Korea; E-Mail: sunmk@inha.ac.kr; 5 Biohybrid Systems Research Center, Inha University, Incheon 402-751, Korea

**Keywords:** biomimetic membranes, lipid bilayer, ion channel sensors

## Abstract

Synthetic biomimetic membranes provide biological environments to membrane proteins. By exploiting the central roles of biological membranes, it is possible to devise biosensors, drug delivery systems, and nanocontainers using a biomimetic membrane system integrated with functional proteins. Biomimetic membranes can be created with synthetic lipids or block copolymers. These amphiphilic lipids and polymers self-assemble in an aqueous solution either into planar membranes or into vesicles. Using various techniques developed to date, both planar membranes and vesicles can provide versatile and robust platforms for a number of applications. In particular, biomimetic membranes with modified lipids or functional proteins are promising platforms for biosensors. We review recent technologies used to create synthetic biomimetic membranes and their engineered sensors applications.

## 1. Introduction

Many researchers have attempted to synthetically mimic biological systems for various practical applications. The cell membrane is one of the most fundamental constituents in biological systems, creating the physical boundaries of cells. The major components of cell membranes include amphiphilic lipids, cholesterols, and membrane proteins. Membranes are not only the physical boundary of cells, but also play important roles in transducing signals, sensing environmental conditions, and recognizing and transporting ions/molecules. In the last few decades, much attention has been devoted to shedding light on the functions and mechanisms of cell membranes. In particular, creating biomimetic membrane systems integrated with functional proteins has been of great interest in relation to physiological studies, drug screening, and sensing platforms [1–5].

Artificial membranes are traditionally reconstructed either by painting a lipid solution or by folding two monolayers [6,7]. The lipid conventionally used to create biomimetic membranes is lecithin, isolated from egg yolk. Membranes created using traditional methods are not suitable for biosensor applications due to their fragility and low resistance. In recent years, a number of techniques to create biomimetic membranes together with synthetic lipids and polymers have widened the field of applicability of biomimetic membrane systems, which now includes artificial cells, drug delivery systems, nanoreactors, and water purification [8–16].

The most important components of biosensors are the targets, the bioreceptors that recognize the targets, and the readers that transduce signals from the bioreceptors. In biological systems, cell membranes play central roles as signal transduction media, converting signals from the bioreceptor into recognizable signals [17,18]. Inspired by cell membranes, biomimetic membranes with modified lipids have become useful tools in biosensing applications [19–21]. Biomimetic membranes have been further engineered with antibodies, aptamers, and other functional proteins for biosensors [22–24].

Herein, we illustrate various types of synthetic biomimetic membrane systems and their applications as compartments or templates for sensors as well as other practical uses. This review will be helpful to researchers who seek to design robust sensor platforms with biomimetic membranes and new biosensors. Biomimetic membranes integrated with numerous functional membrane proteins will provide more versatile and tunable platforms, as compared to those of traditional biosensors.

## 2. Synthetic Lipids and Block Copolymers

### 2.1. Head Modified Lipids

Although lipid bilayers made of naturally occurring lipids provide a very suitable environment for transmembrane elements, the chemical and physical instability of a reconstituted bilayer structure is a major concern for further applications with lipid bilayers. In particular, a freestanding planar bilayer is an attractive platform for studying ion channels and engineered sensor applications. Its fragility and short lifetime, however, present hurdles to further developing practical platforms with lipid bilayers. In order to overcome the drawbacks of the freestanding bilayer system, membranes have been reconstructed onto a solid support, which in some cases is tethered on the surface through head group modification. However, bilayers on solid supports have limitations in terms of feasible bilayer applications, such as single molecule sensors, due to difficulties in achieving highly insulating seals and carrying out long-term DC measurements [25,26], complicating single channel measurements.

A freestanding lipid bilayer created over a small aperture in an insulating film mounted between two halves of chambers provides an almost indefinite amount of ions from both reservoirs and accessibility from both sides of the membrane. In order to make a freestanding membrane system more robust, the use of a hydrogel to support its structure was proposed. In early studies, the membrane was made using conventional techniques; a pre-cast hydrogel was subsequently brought into contact with the membrane in an aqueous solution [27,28]. However, the hydrogel supported membrane was not proven to enhance stability. A more recent study reported in situ encapsulation of a freestanding bilayer membrane with hydrogels [26]. Polyethylene glycol (PEG) based hydrogels are used to encapsulate a lipid bilayer upon UV illumination in the presence of photoinitiators. Intimate contact between the lipid bilayer and the hydrogels was achieved by *in situ* polymerization of the hydrogel without interfering with the ion transport [26].

**Figure 1.**
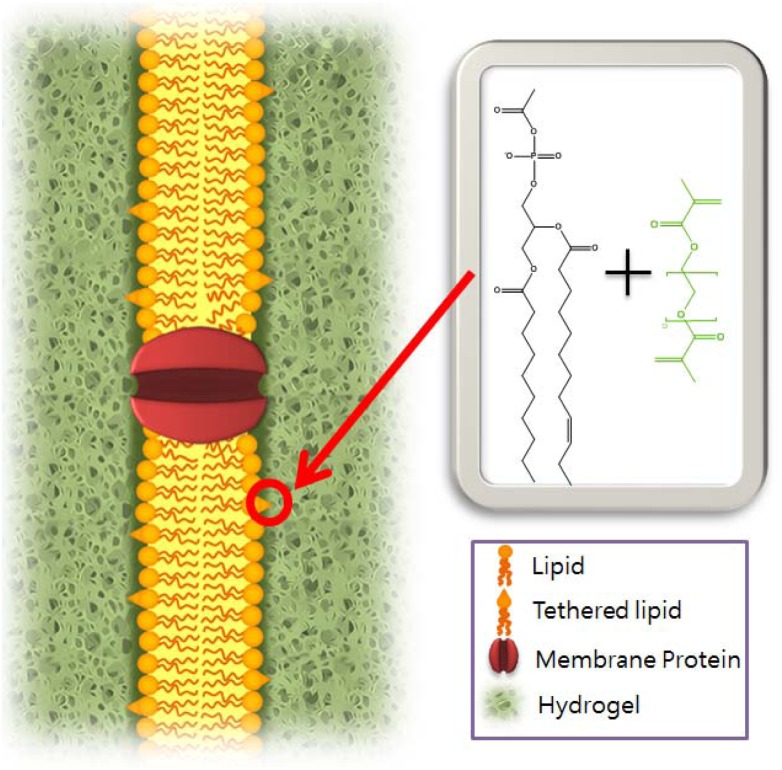
When a hydrogel crosslinks with the head group of a modified lipid, the bilayer is covalently linked to the surrounding hydrogel matrix (right) [29].

To further improve the hydrogel encapsulation method, lipid head groups were modified to provide anchors between the lipid bilayer and the hydrogels [29]. As seen in Figure 1, covalent attachment between the lipid bilayer and hydrogels was accomplished by introducing a crosslinkable double bond at the lipid head group, similar to that in biological systems. Hydrogel conjugated membranes are covalently attached to the hydrogels by crosslinking lipid head groups to the neighboring hydrogels. Chemical reactions of the primary amine at the lipid terminal with N-acryloxysuccinimide yielded crosslinkable head groups. A lipid bilayer was reconstituted over the aperture, as conventionally done [7]. Subsequent UV illumination was used to initiate polymerization of the hydrogels, resulting in attachment between the lipid bilayer and the hydrogels. As a result, the hydrogel conjugated membranes remained intact over a period of days and were stable to mechanical perturbation whereas conventionally made suspended bilayers last up to only 24 h.

A lipid bilayer membrane created with this method became less fluid than a freestanding membrane, while the properties of the ion channels incorporated in the membrane remained unchanged. The decreased diffusivity of the lipid molecules in the conjugated membrane resulted from the covalent attachment between the lipid molecules and the hydrogels. This is similar to the situation in the cell plasma membrane, wherein the diffusivity of lipid molecules is much lower than that in a freestanding membrane [30]. Although the link between lipids and hydrogels in the conjugated membrane decreased the diffusivity of the lipid molecules, ion channels incorporated in the membrane remained functional, as in a conventional freestanding membrane [29].

### 2.2. Block Copolymers

Amphiphilic molecules in an aqueous solution self-assemble into micelles, vesicles, or three other dimensional structures. The amphiphilic molecules found in nature include the phospholipids, cholesterol, and the glycolipids. To mimic a biological structure driven by a self-assembly process, many block copolymers have been designed and synthesized to date. Block copolymers consisting of hydrophilic and hydrophobic blocks aggregate in an aqueous solution, creating micelles and vesicles, as well as various three dimensional structures. Block copolymers are also used as building blocks for freestanding thin films.

#### 2.2.1. Vesicles

**Figure 2.**
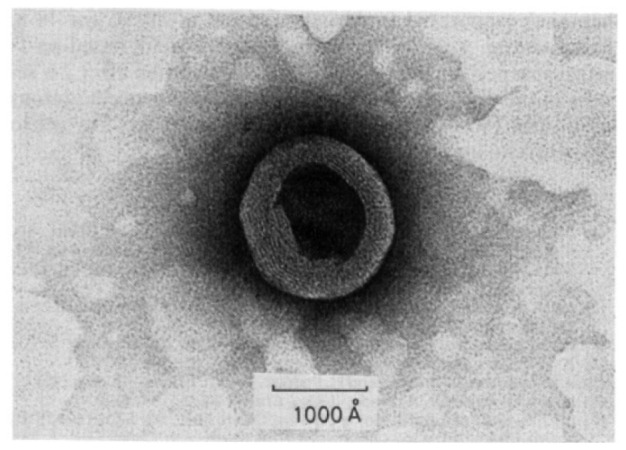
Electron micrograph of didodecyldimethylammonium bromide vesicles. (240,000 X). The sample solution was sonicated in the presence of uranyl acetate. Reprinted with permission from Kunitak *et al.* © 2000 American Chemical Society [31].

Kunitaka and Oksahata reported the first example of synthetic vesicles composed of a simple organic compound [31]. As seen in Figure 2, uranyl acetates in the interior region by hydration with didodecyldimethylammonium bromide blocks result in multilayered vesicles with a diameter of 1,000–2,000 Ǻ. The thickness of the layer of the vesicles is ∼40 Ǻ, both in the lamellar and multi layered form.

**Figure 3.**
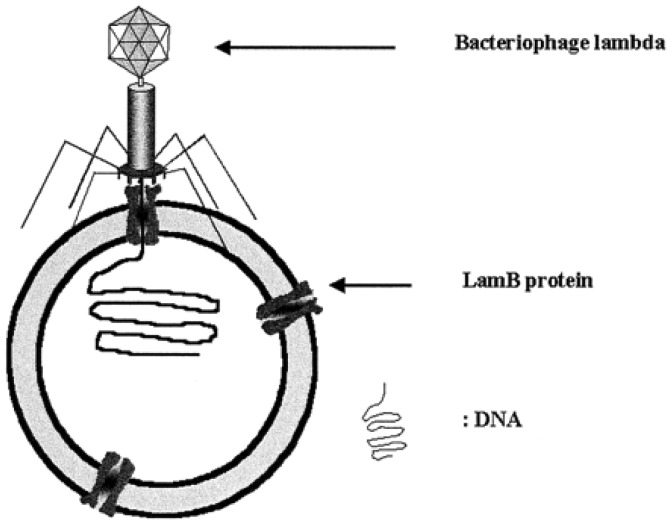
Schematic representation of a DNA-loaded ABA copolymer vesicle. *λ* phage binds to a LamB protein, and the DNA is transferred across the block copolymer membrane. Reprinted with permission from Graff *et al.* Copyright (2002) PNAS [32].

Polymeric membranes in vesicle form have been further studied with various transmembrane proteins. In vesicles made from an ABA triblock copolymer, transmembrane proteins, such as LamB [32], OmpF [33], maltoporin [34], and aquaporin [35], integrated into the membrane, remained functional, as in the conventional membrane structure. Block copolymers have been further engineered for drug and gene delivery systems. Graff *et al.* demonstrated that the functionality of LamB protein is fully preserved in an artificial membrane [32]. Other membrane proteins also remain functional in polymer membranes. As presented in Figure 3, DNA translocation mediated by the *λ* phage receptor LamB was accomplished across a synthetic membrane. The phage-mediated loading of DNA into polymer vesicles presents a plausible model system to investigate the transport of foreign genes into cells. Polymer vesicles incorporated with a few ion channels or pore proteins will generate a number of applications due to the unique characteristics of ion channels. Studies on the incorporation of functional channel proteins into polymersomes and their potential applications are actively ongoing [36]. Other applications with polymer vesicles as either nanocontainers or nanoreactors are discussed later in this review.

#### 2.2.2. Freestanding Membranes

**Figure 4.**
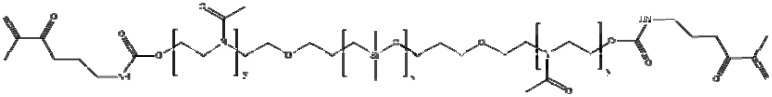
The triblock copolymer PMOXA-PDMS-PMOXA. The central siloxane region mimics the hydrophobic center in lipid membranes while the hydrophilic PMOXA end blocks mimic polar lipid head groups.

The exceptional stability of the block copolymer membranes tested in vesicle form has drawn the attention from researchers investigating freestanding membranes as a potential substitute to biological lipid bilayers. When block copolymers are spread over a small aperture, the molecules self-assemble into a thin film. Amphiphilic block copolymers consisting of hydrophilic and hydrophobic blocks have been proposed to generate mechanically robust self-assembled structures [37–41]. Nardin *et al.* were the first to describe a freestanding monolayer film using a poly(2-methyloxazoline)-*block*-poly-(dimethysiloxane)-*block*-poly(2-methyloxazoline) (PMOXA-PDMS-PMOXA) triblock copolymer (Figure 4). They reported that a physically robust membrane structure is achievable with block copolymers due to the larger size of the hydrophobic blocks as compared to those of lipids. Membranes created on a sub-mm sized aperture showed electrical properties similar to those measured in a lipid bilayer membrane. Furthermore, the triblock copolymer used in their work has a crosslinkable functional group at both ends of the molecule, resulting in a more robust structure through conjugation of neighboring molecules in the thin film.

**Figure 5.**
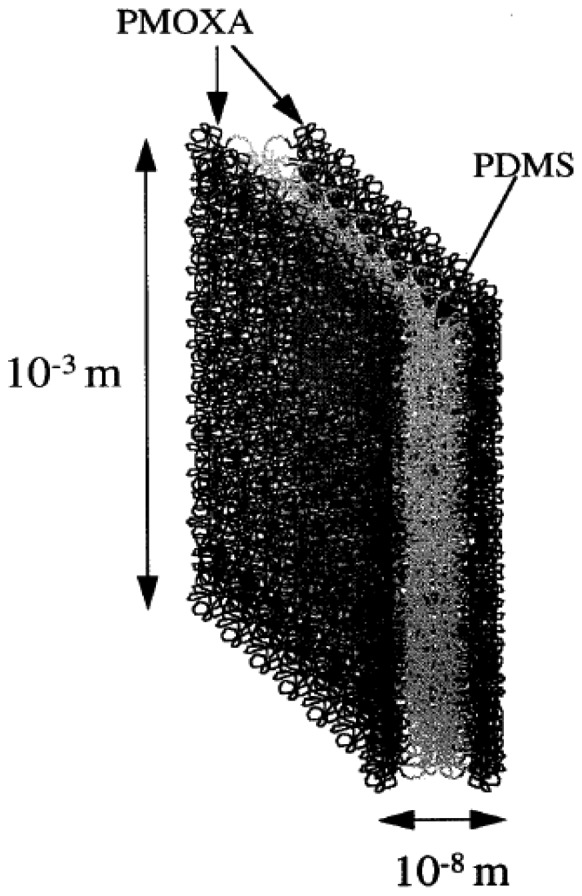
Schematic representation of a planar polymer membrane made from tri-block copolymers: PMOXA (poly methyloxazoline)-PDMS (poly dimethylsiloxane)-PMOXA (poly methyloxazoline). Reprinted with permission from Nardin *et al.* © 2000 American Chemical Society [44].

In a subsequent study shown in Figure 5, Nardin *et al.* demonstrated the first direct measurement of ion channels embedded in a freestanding polymer membrane [44]. A bacterial porin OmpF (outer membrane protein F) was incorporated into a synthetic polymer membrane. When the OmpF was reconstituted into a natural lipid bilayer, it showed a characteristic conductance of 2.1 nS in 1M KCl [42]. The conductance of OmpF incorporated into the polymer membrane was ∼6 nS, indicating either simultaneous insertion of three OmpF or the conformational change of the protein in the polymer membrane. Freestanding block copolymer membranes were further studied by Wong *et al.* Various proteins as well as small channel forming peptides were incorporated into a freestanding ABA block copolymer membrane [43], showing the potential of synthetic polymer membranes as lipid membrane analogues.

### 2.3. Block Copolypeptides

Lipids are among the most biocompatible materials that self-assemble into spherical vesicles in an aqueous environments [45]. Although lipids have been engineered to generate stable platforms in a number of pharmaceutical applications, their mechanical stability and longevity are major concerns. In order to address the shortcomings of lipid based vesicles, synthetic polymeric systems, such as amphiphilic block copolymers, are employed to generate a biocompatible structure that has enhanced robustness and longevity, and yet is biologically active. Among many synthetic block copolymers, polypeptides are promising building blocks that can assemble into various three dimensional structures with high versatility. For instance, modification of peptides with one or more hydrophobic tails results in various arrangements of amphiphilic polypeptides in a configuration of alpha-helices, beta-sheets, and fibers [46]. Such engineered polypeptides have been successfully employed to produce various structures including spherical aggregates, wormlike micelles, and vesicles [47], and these conformations can be reversibly shifted in response to stimuli.

In a biological system, proteins in virus capsids assemble into vesicle-like structures, driven by appropriate folding processes and stable conformations of each subunit [48]. Similarly, it has been recently demonstrated that a hydrophobic polymer peptide in an α-helical conformation undergoes a self-assembly process into vesicles by the ordered conformations of the polymer segments [49,50]. Naturally occurring L-leucine segments were used to prepare hydrophobic domains with stable secondary conformations. Depending on the chain length of each segment of diblock copolypeptides, the polypeptides form various structures including spherical vesicles, sheet-like membranes, and irregular aggregates. Vesicles made of polypeptides provide a versatile platform with abundant functional groups of amino acids, giving rise to many opportunities for drug delivery and sensor applications. For instance, polypeptide vesicles can release encapsulated components by an external stimulus such as pH or temperature.

**Figure 6.**
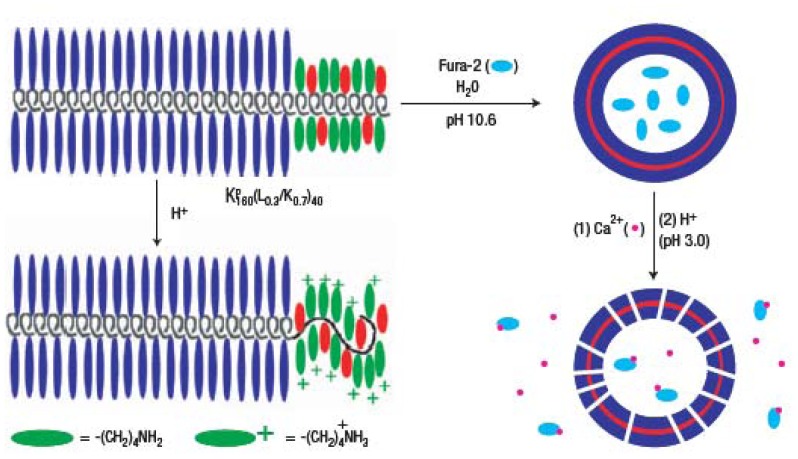
pH responsive polypeptide vesicles. Schematic illustration of a polypeptide, K^P^_160_(L0.3/K0.7)40, and its assembly to a vesicle. Due to the conformational change with pH, molecules entrapped in the vesicle are released. Reprinted by permission from Macmillan Publishers Ltd: Nature Materials [49], copyright 2004.

Bellomo *et al.* demonstrated controlled release of small molecules by replacing 70% of the hydrophobic domain of a diblock copolymer with pH tunable components such as L-lysine(K) [49]. Since the L-lysine domain remained uncharged and water insoluble at high pH, the L-lysine domain neither disrupted the hydrophobicity or disturbed the highly ordered assembly of the chains. However, the hydrophobic domain becomes unstable at low pH due to the charged L-lysine, as can be seen in Figure 6. Recently, Holowka *et al.* [51] addressed the major drawback of peptide based polymeric vesicles. In order to increase their biofunctionality and thereby allow interaction with cells and tissues, polyarginine segments were introduced to the block copolypeptide, providing functionality for efficient drug delivery.

Vesicles made of polymeric materials are usually very stable. Bellomo *et al.* reported that the entrapped dyes in polypeptide vesicles were not released during a period of a few weeks and the hydrophobic wall of the vesicles was not disrupted by high temperature (up to 90 °C), time (six months), or harsh pH conditions, thus showing superior stability and longevity over conventional lipid based vesicles. Owing to their increased stability, stimuli responsiveness, and chemical versatility polypeptide vesicles are a promising platform to provide robust encapsulants for pharmaceutical applications.

## 3. Sensors and Other Engineered Applications

Biomimetic membranes provide an optimal environment for transmembrane elements. However, there remain a number of technical challenges to meet before it is possible to realize membrane sensor applications. In order to use biomimetic membranes as a robust platform for biosensors, the following requirements should be met: (1) Membranes should be robust and preferably storable. (2) To measure the small conductance of ion channels, the membrane should maintain high resistivity, enabling channel measurements at the single molecule level. (3) An array format of membranes should be considered so as to allow miniaturization of the sensor chip, enabling parallel sensing or high-throughput measurement. (4) Sensors should be designed to be used by unskilled users. User friendly interfaces for measurements and data analysis should be considered.

Although numerous proof-of-concept biosensors using a biomimetic membrane platform have been introduced to date, numerous hurdles must be crossed in ordered to advancing the technology to the level of practical applications. However, recent technological improvements are approaching these requirements. Herein, some biosensor applications using biomimetic membranes either in a planar membrane configuration or a vesicle configuration are introduced.

### 3.1. Supported Membranes

Planar lipid membranes are the most attractive platform for biosensor and drug screening applications despite their fragility and instability. Numerous studies focused on addressing the shortcomings of planar membranes have been published to date [52,53]. Supported bilayers have been suggested as a means of improving the stability of lipid membrane structure and these bilayers have been well characterized.

**Figure 7.**
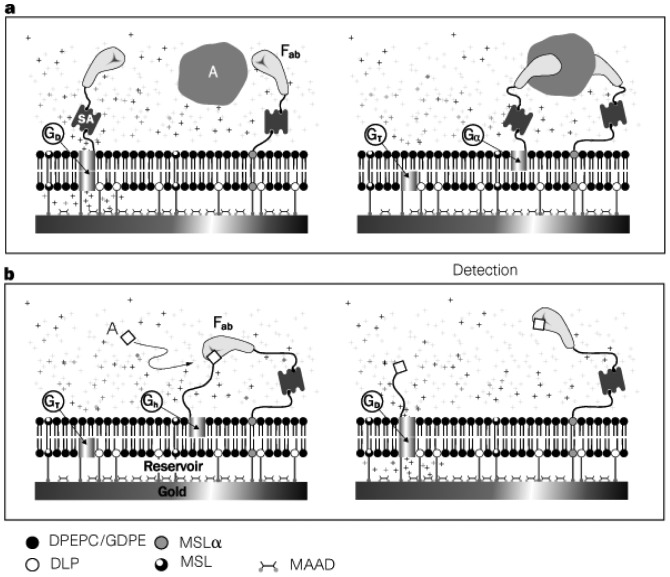
(**a**) Two-site sandwich assay. Immobilized ion channels (GT), synthetic archaebacterial membrane-spanning lipids (MSL) and half-membrane-spanning tethered lipids (DLP) are attached to a gold surface. In addition, the membrane is composed of mobile half-membrane-spanning lipids (DPEPC/GDPE) and mobile ion channels (G). The mobile ion channels are coupled to biotinylated antibody fragments (Fab') using streptavidin (SA). When the target analyte is approached to the bilayer, the gramicidin A dimer is displaced upon the binding of the analyte to the binding sites of the lipid surface and of gramicidin, decreasing impedance across the bilayer. (**b**) Competitive assay. The membrane contains hapten-linked gramicidin, (Gh). When the addition of analyte competes with the happen for Fab', hapten-linked gramicidin will be liberated and associated with another gramicidin, increasing impedance across the bilayer. Reprinted by permission from Macmillan Publishers Ltd: Nature [54], copyright 1997.

The most influential work was conducted by Cornell and coworkers, who developed ion channel biosensors using a tethered lipid bilayer on a solid surface [54]. The tethered bilayer was used as ion channel sensor platform. Tethered bilayers usually have resistance that is orders of magnitude lower than that of freestanding bilayers [55,56], thus limiting the applicability of ion channels as signal transducers to develop biosensors with high sensitivity. In order to ameliorate the low resistance of a tethered membrane, Cornell and coworkers used various phytanyl lipids having branched methyl groups in the hydrocarbon tails. In particular, synthetic archaebacterial membrane-spanning lipids (MSL) was used to anchor lipid molecules on a gold surface, leading to improved resistance. As illustrated in Figure 7, dimeric gramicidin A channels are dissociated from each other when the target molecule is introduced to the binding sites of the lipid surface and of gramicidin, resulting in decreased conductivity across the membrane. Similar principle was applied to competitive assay shown in Figure 7(b). Target molecules can be sensed by measuring impedance across the membrane in a highly sensitive manner.

**Figure 8.**
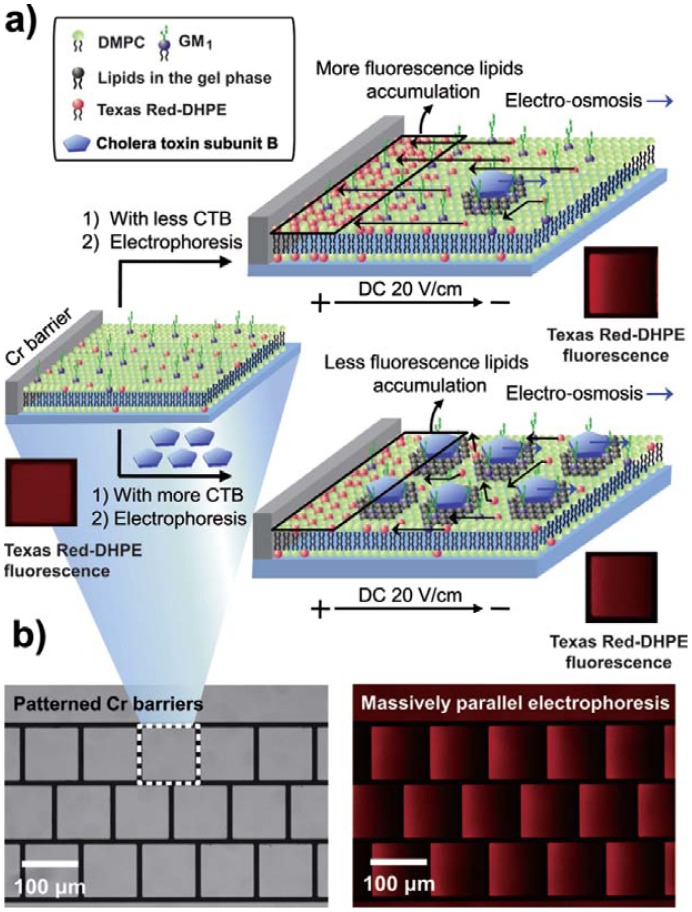
(**a**) Schematic illustration of an electrofluidic lipid membrane biosensor. Red insets are microscope images of the SLB. (**b**) Left: microscope image of multiple corrals patterned on a glass substrate. Right: electrophoresis of multiple SLB patterns. Reprinted with permission from Lee *et al.* Copyright (2012) John Wiley & Sons, Inc. [58].

More recently, supported lipid bilayers have often been classified into several categories, including solid-supported lipid bilayers (SLBs), tethered lipid bilayers (tSLBs), and polymer cushioned lipid bilayers (pSLBs). Recent advancements in technology have allowed wider use of tSLBs [57]. A lipid bilayer itself can serve as a sensing platform with no functional membrane proteins [58]. The electrofluidic lipid membrane biosensor, shown in Figure 8, can enable the detection of cholera toxin subunit B with a lipid-tethered ganglioside (GM1). SLB is composed of dimyristoylphosphatidyl-choline (DMPC), GM1, and negatively charged dihexadecanoylphosphatidylethanolamine (DHPE) conjugated with Texas Red. DHPE-Texas Red migrates toward a positively charged electrode upon the application of an electrical field. When a toxic substance binds to GM1, a fluid to gel transition takes place in lipids around GM1, which interferes with the migration of DHPE-Texas Red under certain electric fields. The concentration of the toxic substance can be measured by analyzing the fluorescent gradient derived from the migration of DHPE-Texas Red.

### 3.2. Surface Plasmon Resonance

**Figure 9.**
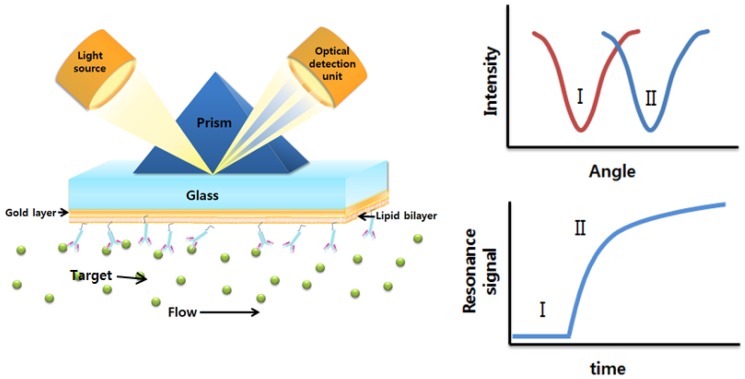
Schematic illustration of SPR and a typical sensorgram. A sensor chip measures the intensity of the reflection of the incident light due to the interaction between the target molecules (green spheres) in the flow solution and the probe molecules (pink diamonds).

Surface plasmon resonance (SPR) spectroscopy measures changes in the refractive index of a sensor chip. The principle of SPR is based on the Kretschmann theory of the resonance of surface plasmons, published in 1971 [59,60]. When light is shone on an interface, energy from the photons of light is transferred to free electrons on the interface to generate collective excitations, called surface plasmons [61]. Figure 9 shows a typical SPR setup based on the Kretschmann configuration. A metal layer, usually gold, is evaporated onto the glass. The light shone on the prism is totally reflected. Despite the total reflection, a small amount of light, evanescent waves, will still penetrate the interface of the two materials, resulting in collective excitation by the energy transfer from the photons of the light into the interface.

The waves on the surface are highly sensitive to the properties of the interface, resulting in changes to the refractive index. Changes made on the interface result in a shift in the resonant wavelength of the incident light, the intensity of the reflected light, or the resonant angle of the incident light [61]. SPR spectroscopy can be used to characterize the surface phenomena by measuring the magnitude of this shift of the surfaces. When target molecules bind to the surface through immobilized probes or receptors the binding events and conformational changes made on the target molecules or probe molecules can be measured with high sensitivity. This regime has been applied to a number of biological applications.

SPR spectroscopy is combined with impedance measurements, which are conventionally used to test molecular assemblies at a solid surface [62]. Together with electrical measurement, in situ transmembrane interaction with ligands can be monitored with high sensitivity by detecting changes of the optical parameters in a solid liquid interface. Commercially available SPR spectroscopy makes it possible to perform a binding assay with membrane proteins [63]. Although binding kinetics can be well characterized by this method, additional measurements are required to obtain physiologically meaningful data. In order to study binding kinetics and their correlations with the function of channel proteins, a tethered bilayer is used to enable simultaneous measurements using both impedance and SPR spectroscopy. It allows researchers to probe the binding of the ligands to the ion channels and the subsequent changes of the channel activities [64].

**Figure 10.**
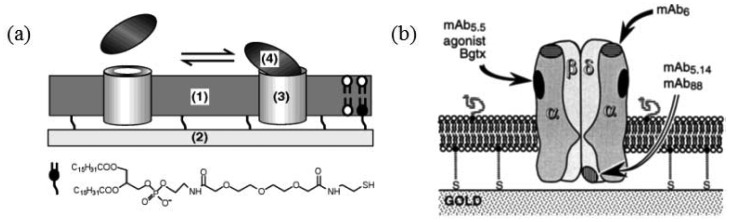
(**a**) Schematic illustrations of an OmpF (3) containing lipid bilayer tethered to a solid surface (2) and ligand interaction with the TR domain of colicin N. Reprinted with permission from Stora *et al.* [64] Copyright (1999) John Wiley & Sons, Inc. (**b**) nAchR reconstituted in a tethered bilayer: determining receptor orientation and functionality. Reprinted from Sévin-Landais *et al.* [66] Copyright (2000) with permission from Elsevier.

In addition, micropatterned lipid bilayers integrated with membrane proteins using head modified lipid molecules provide robust platforms, enabling the measurement of interaction between membrane proteins and their specific ligands as shown in Figure 10. G protein-coupled receptors (GPCR) are the most abundant receptors of high physiological interest. Sensory receptors, such as taste receptors, have most representative functions in physiological systems [65]. Combined with recent microfabrication techniques, automated SPR spectroscopy may provide a high-throughput platform to study ion channels. In addition to pharmaceutical purposes, a lipid bilayer integrated with membrane proteins provides a highly-controlled environment, enabling biosensor applications with high sensitivity and specificity.

### 3.3. Colorimetric Assay Using PDA Vesicles

The use of a vesicle as a sensing platform offers many advantages due to its functionality and applicability to numerous biological systems. In particular, polydiacetylene (PDA) vesicles are considered attractive platforms owing to their versatility to introduce various functional groups on the vesicle surface. Vesicles made of PDA undergo a colorimetric transition that can be seen by the naked eye, enabling label-free and rapid sensing platforms.

**Figure 11.**
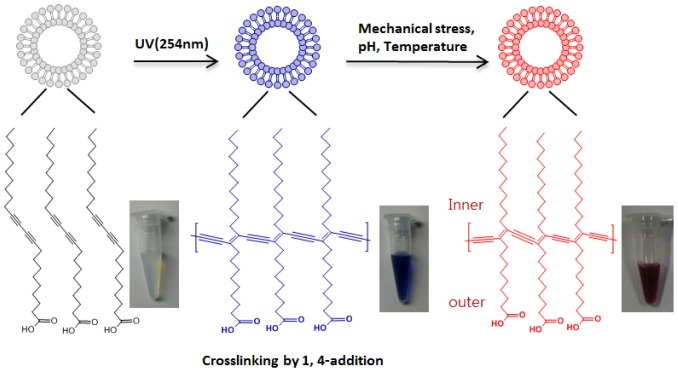
Chemical structure of polydiacetylene and its chromatic properties. (**a**) Polymerized backbone of vesicles. (**b**) blue-red color transitions (drawn with ChemDraw, PerkinElmer, Inc.).

Figure 11 shows representative diacetylene molecules with carboxylic acid terminals, to which sensing elements, such as antibody and aptamers, can be conjugated. The lipid-like amphiphilic structure with a methyl terminated hydrophobic tail and a hydrophilic carboxylate head group drive self-assembly into either sheets or vesicles. The crosslinkable diacetylene backbone of each molecule is aligned by a self-assembly process in an aqueous solution, enabling polymerization with an adjacent molecule. Polymeric vesicles can be crosslinked through 1,4 addition of aligned diacetylene monomers [67]. The polymerization reaction initiated by UV irradiation results in a robust structure whereas liposomes are often subject to short lifetime due to oxidation and mechanical instability.

The most attractive feature of vesicles made of polydiacetylene is their capability of rapid colorimetric transitions by environmental stimuli including pH, temperature, and pressure [68,69]. Polymerization results in a color transition of vesicles to deep blue. This color transition is attributed to electronic delocalization within the conjugated framework, resulting in increased absorption at 650 nm, which appears as a deep blue color [70]. Polymerized diacetylene can undergo rapid blue to red color transitions by various perturbations. The conformational changes of the polymeric backbone are known to cause the blue to red color transition. Most common polydiacetylene derivatives usually undergo color transitions in an irreversible fashion. However, recent studies have shown that reversible color changes can be achieved through modification of the head groups of PDA [71–73], thus broadening the applicability of PDA based supramolecules in diverse analytical and diagnostic areas.

**Figure 12.**
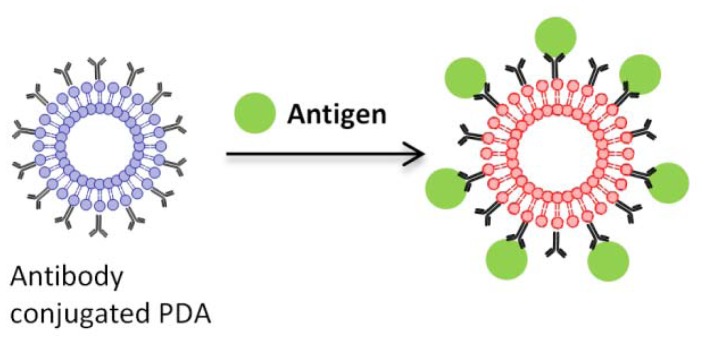
Blue to red color transition upon ligand binding to the receptor conjugated to the head group of PDA (drawn with ChemDraw, PerkinElmer, Inc.).

Various modifications of the head group enable many attractive sensor applications. The polymerizations are initiated by UV irradiation, creating a robust membrane or vesicle structure. The most common biosensor application with PDA vesicles is molecular recognition using receptors. Figure 12 shows the modification of a PDA head group with recognition elements. The recognition elements attached to the surface of the PDA vesicles are easily accessible to ligands and provide abundant recognition sites. Upon binding of specific ligands to their corresponding recognition sites, rapid blue to red color changes occur. The blue to red transitions are attributed not only to physical changes on the surface, but also to other perturbations.

To quantify a colorimetric transition, the parameter denoted %CR (percentage colorimetric response) is defined as given in the following equation:

$$CR=\frac{PB_{0}-PB_{I}}{PB_{0}}\times 100\%$$

where $PB=\frac{A_{640nm}}{A_{640nm}+A_{550nm}}$. A is the absorbance of either the blue or the red component, which peaks at 640 nm and 550 nm, respectively. *PB*_0_ is obtained before the color transition and *PB*_I_ is a measured value after the color transition. A higher CR value indicates that a stronger blue-to-red transition is made.

Lipid-like amphiphilic assembly of PDA enables the creation of hybrid vesicles composed of a mixture of synthetic polymers and lipids, in which synthetic and biological constituents can be incorporated. For instance, early work with PDA/lipid hybrid vesicles enabled the realization of ion selective sensors using ionophores. The PDA/lipid hybrid vesicles integrated with ionophores showed selective colorimetric responses to ions with different affinities [74]. Aside from ion sensors, a number of different sensors can be constructed using various molecular recognition entities. Representative biosensors using PDA vesicles, as developed to date, include PDA microarray sensors on solid substrates [75,76], microbead-assisted PDA sensors [23], PDA-embedded electrospun fibers [77], encapsulated PDA sensors [78], and microfluidic PDA sensors [79,80].

### 3.4. Nanocontainers

**Figure 13.**
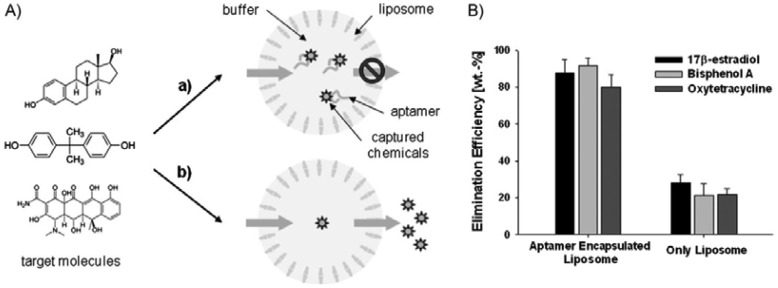
(**A**) aptamer-in-liposome composite (a) and liposomes without aptamer (b). (**B**) Chemical capturing efficiency of the aptamer-in-liposome composite and a liposome without an aptamer encapsulated for three small organic compounds. Reprinted with permission from Kim *et al.* Copyright (2011) John Wiley & Sons, Inc. [88].

Liposomes made of synthetic lipids or polymers are often used as biomimetic materials for drug delivery and biosensor applications [81–86]. In order to deliver drugs to a specific target, encapsulants should be contained in proper environmental conditions. Cavities created in liposomes are the best site for drug storage due to their stability and tunability [87]. Liposomes with multiple encapsulants can be readily created in various sizes from a few hundred nanometers to tens of micrometers. Figure 13 shows a recent work with aptamers-in-liposome composites that contain specific aptamers to capture small compounds [88]. The aptamers encapsulated in liposomes remain stable in a well-protected cavity regardless of the surrounding environment. The results shown in this work imply that the small space in the liposomes will help sustain the binding affinity of aptamers, together with an appropriate binding buffer. The small molecules that pass through the liposome will be specifically bound to aptamers encapsulated in the liposomes, while the aptamers remain within the liposome, resulting in increased capture efficiency. Aside from this aptamers-in-liposome structure, a number of applications can be devised due to the versatility of liposome nanocontainers with various encapsulants. More recently liposome nanocontainers have been applied to photosynthesis [8], artificial cells [9], and ATP biosynthesis [89].

Similar to the case of lipid vesicles, synthetic polymers that self-assemble to vesicles have recently been employed as nanocontainers in which multiple molecules are encapsulated [90–92]. Of particular interest, block copolymers have been proven to be well-suited amphiphilic molecules, resulting in vesicles in which proteins or enzymes can be encapsulated [93,94]. Aside from sensor applications, nanocontainers are considered an ideal platform for drug delivery systems due to their high stability and longevity [95]. Polymer vesicles made of block copolymers such as PMOXA-PDMS-PMOXA are impermeable to water and other molecules [91]. Therefore, these polymersomes provide versatile platforms, including nanocontainers and ion channel sensors.

**Figure 14.**
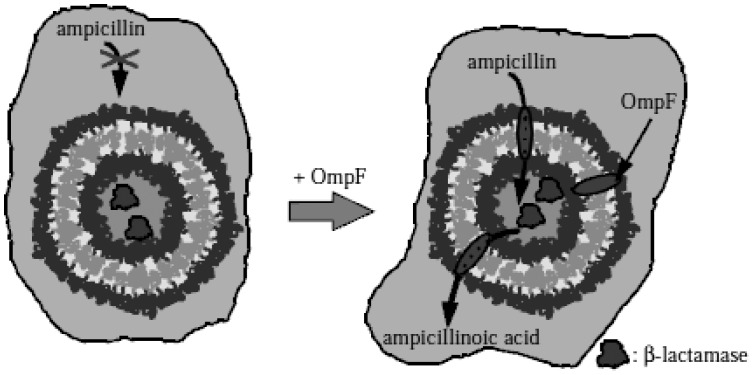
PMOXA-PDMS-PMOXA polymersome with encapsulated β-lactamase. Left side: ampicillin cannot permeate polymersome membrane. Right side: ampicillin and ampicillinoic acid can transfer across the polymersome membrane through OmpF channel. Reprinted with permission from Nardin *et al.* Copyright (2001) John Wiley & Sons, Inc. [96].

Nardin *et al.* created nanoreactors integrated with channel proteins [33]. First, β-lactamase was encapsulated by the nanoreactor. To release the β-lactamase in a controlled manner, they used a well-characterized membrane channel, the bacteria porin OmpF (outer membrane protein F). The functionality of the membrane protein was shown to be fully preserved within the synthetic surroundings. The same research group demonstrated another application, as shown in Figure 14. Ampicillin molecules do not permeate through the membrane of the polymersome, whereas OmpF facilitates permeation of ampicillin through polymersomes [96].

## 4. Conclusions/Outlook

The lipid bilayer is a major constituent of the cell membrane that physically separates biological systems from the outer world. There are also thousands of proteins associated with the cell membrane. These proteins play important roles in many biological functions including sensing the environment, signal transduction, and transportation of various ions and molecules across the membrane, which are critical for sustaining cellular vitality. In efforts to enhance our understanding of the molecular basis functions of biological membranes and membrane associated proteins, various artificial membrane systems have been developed and used as model systems to elucidate protein and membrane biochemical characteristics. These artificial membranes integrated with functional proteins are of particular interest for drug screening and sensor applications, since the molecular recognition events of membrane proteins against target molecules transpire in a highly specific manner. For these purposes, lipid membranes are typically reconstructed in planar or vesicle form. However, their intrinsic instability has emerged as a major hurdle that must be overcome before commercial application of given systems. To address this challenge and improve the robustness of the membrane, we have provided strategies that have been proposed: (1) hydrogel supported membranes, (2) membranes made of amphiphilic block copolymers or block copolypeptides with longer hydrophobic tails, (3) supported membranes on a solid surface. Aside from these strategies, many other attempts have been made to date. These biomimetic membranes introduced in the review are fully functional in terms of insulating properties and provide an appropriate environment for incorporated proteins to retain full activities. The improved stability of biomimetic membranes makes it possible to integrate them with electrochemical or optical sensor architectures, further broadening their analytical spectrums. The miniaturization of sensing elements and the production of membranes in array form are the directions of future development for both field applications and multiplex analysis. Combining a sophisticated microfluidic platform and an array based system, and with current advances in detection technologies, we should see the development of more accurate and higher throughput analytical systems. For instance, these platforms will be able to provide powerful tools for the discovery of novel drugs, for clinical diagnosis, as early warning sensors for biologically and chemically hazardous compounds in food and the environment, and various single molecule based studies. Perhaps the ultimate goal of biomimetic artificial membrane technology will be the realization of cell-like systems. By elaborately combining a series of engineered proteins with the membrane it will be possible to construct powerful synthetic machinery for smart drug delivery and miniature bioreactors, and self-functional sensing systems.
